# *Treponema pallidum* TprD and TprK are adhesins and their surface expression promotes spirochetal opsonophagocytosis

**DOI:** 10.3389/fimmu.2026.1783902

**Published:** 2026-03-06

**Authors:** Kashaf Zafar, Onyedikachi C. Azuama, Linda H. Xu, Lorenzo Giacani, Nikhat Parveen

**Affiliations:** 1 Department of Microbiology, Biochemistry and Molecular Genetics, Rutgers-New Jersey Medical School, Newark, NJ, United States; 2 Division of Allergy & Infectious Diseases, Department of Medicine, University of Washington, Seattle, WA, United States; 3 Department of Global Health, University of Washington, Seattle, WA, United States

**Keywords:** adherence, opsonophagocytosis, syphilis, TprD, TprK, *Treponema pallidum*

## Abstract

**Background:**

*Treponema pallidum* subspecies *pallidum* causes systemic syphilis, exclusively infects humans in nature and can persist for decades in the absence of treatment despite generating robust adaptive immune responses. The *T. pallidum* repeat (Tpr) family of outer membrane proteins are immunogenic and are implicated in immune evasion, indicating them to be virulence factors displayed on the spirochete surface. Long-term survival of *T. pallidum* is largely attributed to sparse surface-exposed outer membrane proteins and antigenic variation in the major surface protein TprK through phase variation. This mechanism has been studied for decades; however, the functions of Tprs of this extracellular pathogen are not yet experimentally determined.

**Methods:**

In this study, the localization and functional roles of TprD and TprK were investigated using a heterologous spirochete expression system and a gain-of-function approach by employing a non-infectious, non-adherent *Borrelia burgdorferi* B314 strain. Opsonophagocytosis of engineered *B. burgdorferi* as well as of *T. pallidum* Nichols and SS14 strains was evaluated using J774A.1 macrophages and mouse antibodies raised against predicted surface-exposed loops of TprD and TprK using IncuCyte system.

**Results:**

Both TprD and TprK were found to be surface exposed in engineered *B. burgdorferi* and infectious *T. pallidum* strains. Expression of these proteins conferred adherence to several mammalian cell lines *in vitro*. In addition, antibodies we generated recognized TprD and TprK on the surface of spirochetes and significantly enhanced macrophage-mediated opsonophagocytosis.

**Conclusion:**

Our findings here demonstrate that TprD and TprK function as *T. pallidum* adhesins that are also targets of opsonic antibodies. These Tprs likely facilitate tissue colonization during infection, while also rendering the pathogen susceptible to immune clearance. Our findings support inclusion of TprD and TprK as components of a multivalent vaccine against syphilis.

## Introduction

*Treponema pallidum* subspecies *pallidum* (*T. pallidum*) spirochetes are the causative agent of syphilis, which is a re-emerging global health problem characterized by systemic infection and lifelong disease in the absence of treatment. The World Health Organization (WHO) estimates that 5.6–11 million new syphilis infection cases arise among men and women aged 15–49 years annually, with a prevalence of 18–36 million cases globally (1–5). In addition, congenital syphilis (CS), caused by transplacental transmission of *T. pallidum* during pregnancy, has been increasing around the world, even in the Western countries. Vertical transmission of *T. pallidum* is a significant cause of stillbirth, neonatal death, and CS in newborns associated with severe neurologic and developmental defects with estimated ∼700,000 cases reported per year (6–14). The increase in CS to 3,882 cases in the United States in 2023 and a further 2% surge in 2024 underscores the shortcomings in disease control even in high-income nations (15–17). HIV transmission increases by 2–5 times in symptomatic syphilis patients (18, 19), further making *T. pallidum* infection a global public health problem.

The genome of *T. pallidum* has a relatively small size of ∼1.14 Mbp encoding >1000 proteins (20). In nature, it is an exclusively human pathogen. *T. pallidum* pathogenesis and persistence is attributed mainly to the architecture of its outer membrane, with few and poorly displayed surface proteins, antigenic variation in TprK, and stochastic expression of some surface proteins (21–25). By limiting the density of its surface antigens, this pathogen becomes difficult for the host immune system to detect and eliminate from host (26, 27). The characterization of the molecular basis of host-*T. pallidum* interactions and the understanding of syphilis pathogenesis remain rather challenging due to the fragility of spirochete outer membrane, inability to cultivate it continuously in pure culture *in vitro*, and difficulty in generating mutants (28, 29) albeit recent advances in inserting antibiotic cassettes and reporters into the *T. pallidum* genome (3). Therefore, the functional assessment of *T. pallidum* virulence factors has currently been limited to the application of recombinant proteins and surrogate systems (25, 30–36).

In contrast to *T. pallidum*, the structurally and physiologically related Lyme disease-causing spirochete, *Borrelia burgdorferi*, with a genome size of ∼ 1.52 Mb expresses approximately 132 surface lipoproteins, which include known outer surface proteins (Osps) and other virulence factors (37–42). The majority of surface proteins of *B. burgdorferi* are highly immunogenic and dynamically regulated in response to colonization and survival in diverse environments (e.g., tick vs. mammalian host) and are encoded mostly by genes located on endogenous circular and linear plasmids that can be lost during long-term *in vitro* cultivation, rendering spirochetes non-infectious (43). To overcome the challenges that hinder direct functional studies with physiologically active *T. pallidum*, we previously developed a heterologous spirochete expression system that enables the assessment of the subcellular localization of *T. pallidum* proteins and allows evaluation of their functions. We have successfully used a non-infectious and non-adherent derivative of the *B. burgdorferi* B31 strain, B314, as a surrogate to examine the role of some *T. pallidum* proteins (25, 34–36, 44). B314 is a highly passaged strain that has lost various endogenous plasmids and virulence functions, rendering it useful as a heterologous expression system to investigate adherence mechanisms of other pathogenic spirochetes. This gain-of-function approach has contributed toward elucidating structural components of *T. pallidum*, in characterizing the function of some *T. pallidum* surface proteins and validating their potential as vaccine candidates (25, 35, 45). Although the mechanisms of *T. pallidum* persistence have not been completely elucidated, stochastic expression of *T. pallidum* proteins on the bacterial surface and antigenic variation of prominent TprK protein by phase variation likely facilitate survival despite stimulation of the specific adaptive immune response during infection (25, 46).

The *Treponema pallidum repeat* (Tpr) family includes 12 proteins, some of which are known to be surface-exposed (20, 47, 48). They are predicted to possess β-barrel structures with surface loops exposed to the host immune system and have been regarded as key players in bacterial pathogenesis. They are categorized into three subfamilies according to amino acid homology. Subfamilies I and II contain TprC, D, F, I and TprE, G, J, respectively, possess conserved amino and carboxyl terminal sequences but variable central amino acid sequences, whereas the subfamilies III (TprA, B, H, L, K) possess conserved sequences and scattered variable regions across their length (46, 47). These proteins exhibit homology to the major sheath protein (Msp) proteins of *T. denticola*, which have been previously implicated in cell attachment and also function as porins (47, 49, 50). TprK (encoded by the *tp0897* gene) is a highly expressed surface protein with a putative, cleavable signal peptide, predicted molecular weight of 52.7 kDa and has received considerable attention due to its allelic variation and its perceived role in immune evasion despite generating opsonic antibodies. However, surface exposure of this protein was previously challenged by one research group (47, 51, 52). TprK harbors seven discrete variable regions (V1-V7) that are predicted to form external loops at the host-pathogen interface. Furthermore, previous studies have shown that TprK elicits strong T-cell and antibody responses in the rabbit infection model (35, 47, 53–55).

The role of TprC/D in syphilis infection has also been emphasized by several researchers and these homologous proteins have been shown to be targets of humoral and cellular immune responses (46, 53, 56, 57). The TprC/D (Tp0117/131) are duplicated in the Nichols strain, has outer membrane localization and has been predicted to function as porins (20, 58–60). Although TprC and TprD are closely related, they exhibit differences in regions that may be exposed on the bacterial surface. TprC shows fewer hypervariable regions and is more conserved among different strains; however, TprD/TprD2 exhibit sequence variation among strains (for example, in Nichols vs. SS14 strain), particularly in the external loops. The *tprD2* allele (found in the most currently circulating strains) possesses a 330 bp central variable region (CVR) and three smaller regions toward the end of the ORF that distinguishes it from *tprD*, which is found in the *T. pallidum* reference Nichols, Chicago, and Bal73–1 strains (46, 56, 61). Additionally, the *tprC* locus in Bal 3, Mexico A, Sea 81-4, and Street 14 encodes a TprC protein with fewer amino acid variations when compared to the TprC of the referenced Nichols, Chicago, and Bal 73–1 strain (60). It is important to note that the sequence variations in TprD/C are localized in discrete variable regions. TprC/D paralogs have been reported as strong vaccine candidates in previous studies, where it was shown that the N-terminal conserved region induced strong T-cell and antibody responses during infection. Furthermore, immunization with this specific region of proteins reduced the development of early syphilis lesions when vaccinated rabbits were challenged by the *T. pallidum* strain, indicating their protective roles (56, 61).

For an extracellular pathogen like *T. pallidum*, receptor-mediated adherence to host tissues plays a crucial role in colonization of different organs, and its long-term persistence in hosts. Upon infection, phagocytosis of opsonized treponemes by macrophages aid in the clearance of spirochetes from early cutaneous lesions (62–66); however, this clearance mechanism is rather slow, likely due to limited surface exposure of proteins. The N-terminal region is conserved among all members of subfamily I Tprs, eliciting antibody and T-cell responses. Immunization with this region was reported to minimize the development of syphilitic lesions upon infectious challenge (47, 56). Although TprD exists in two allelic forms: (TprD and TprD2), antigenic variation in these proteins has not been reported to occur during infection by a particular strain. In contrast, TprK undergoes extensive intrastrain antigenic variation during progress of infection (24, 67), via a mechanism based on non-reciprocal gene conversion from a pool of donor sequences. Furthermore, infection-induced antibodies target the V regions of TprK, with sequence diversity localized within the discrete variable regions, rather than the known conserved regions. A previous investigation showed that the convalescent sera recovered from rabbits immunized with recombinant TprK recognize both variable and conserved regions (55).

Antigenic variation is known to be associated exclusively with the surface exposed proteins. By using antibodies generated against putative outer loops of one allele each of both TprK and TprD, we show that these proteins are displayed on the surface of engineered *B. burgdorferi* B314 and on *T. pallidum* Nichols and SS14 strains. These Tpr proteins are also targeted by the specific antibodies to facilitate opsonophagocytosis by macrophages *in vitro*. Although TprD and TprK proteins have been studied for decades, the function of these proteins as adhesins remained undiscovered, although TprK was proposed to be a monomeric porin (68). We carried out this investigation to experimentally show that the selected TprD and TprK proteins are also adhesins like their homolog, Msp of *T. denticola*. We demonstrated the adhesin function of TprD and TprK by expressing them in the non-adherent B314 strain of *B. burgdorferi* which then gained ability to bind to different mammalian cell lines.

## Results

### Antibody production against TprD/TprK

**Figure 1:**
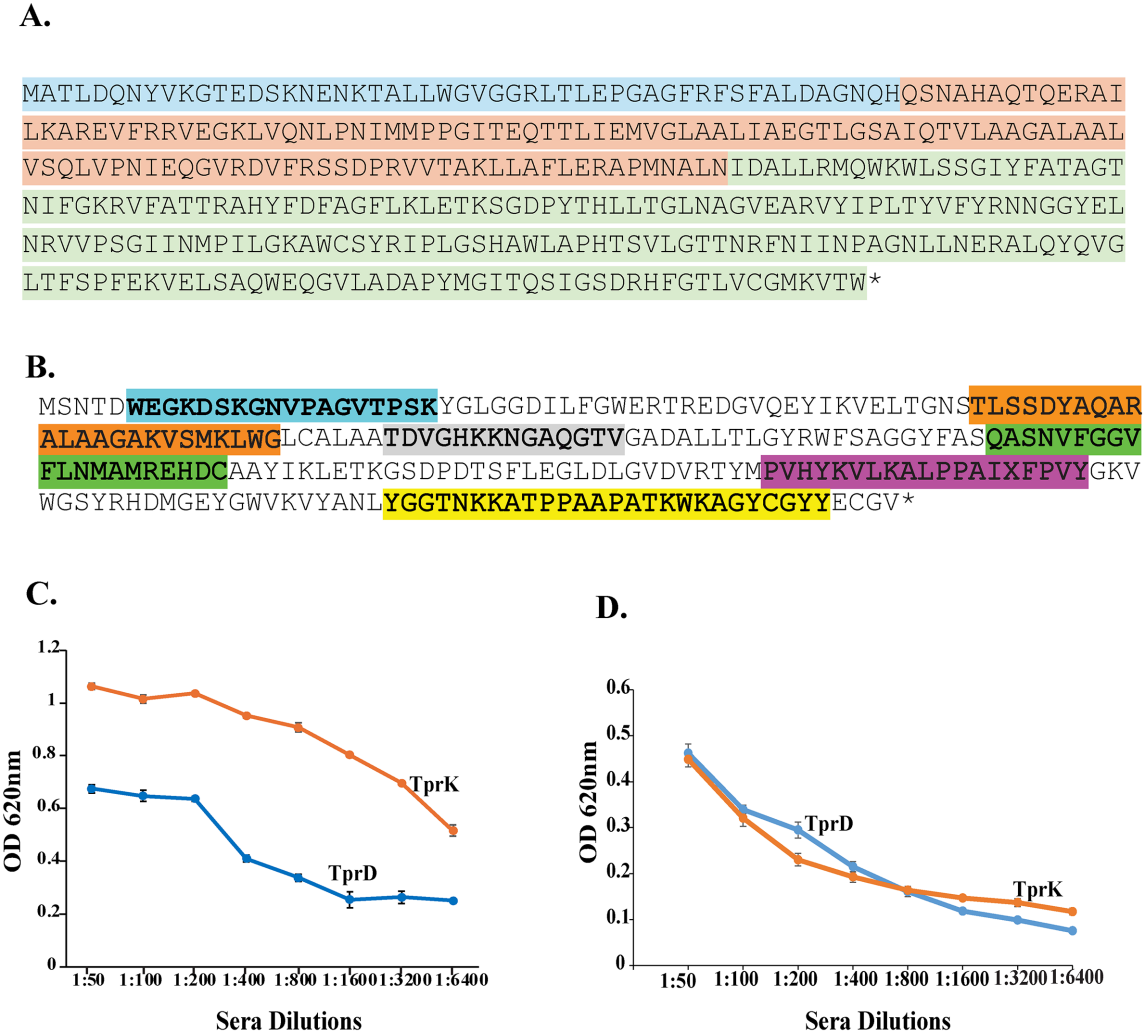
Sequence of Tpr chimera constructs generated by GenScript. DNA encoding TprC/D or TprK chimera was inserted in pET30a vector to express recombinant His-tagged protein and purification by Ni-affinity chromatography. **(A)** Amino acid sequence of the synthetic TprC/D chimera. Previously identified MOSP^N^ region is highlighted in blue, the CVR in orange, and the MOSP^C^ β-barrel domain in green. **(B)** The chimera protein sequence for TprK includes variable outer epitopes with V2 highlighted in cyan, V3 in orange, V4 in gray, V5 in green, V6 in purple, and V7 in yellow color. Sera were titrated against: **(C)** respective full length His-tagged recombinant proteins, or **(D)** B314 expressing TprD or TprK by ELISA.

The protein sequences of TprC/D (**Figure 1A**) and TprK (**Figure 1B**) used for the generation of chimeras were generated by GenScript in pET30a expression vector and confirmed by sequencing. The recombinant proteins purified from *E. coli* were used for Balb/c mice immunization and for different experiments in this study. We determined titer of pooled antisera from five mice for each chimeric protein against full length recombinant TprD and TprK (**Figure 1C**) or intact B314(TprD) or B314(TprK) in wells (**Figure 1D**). Titer of each pooled antisera were higher with the respective recombinant full-length proteins compared to respective proteins expressing intact B314 (**Figures 1C, D**). We also generated clones in pJSB175 plasmid vector containing one allele each of full length TprD and TprK encoding sequences together with flanking DNA sequences that include potential promoter and downstream sequences (**Supplementary Figure 1**). B314 strain transformed by these clones allowed TprD and TprK proteins expression under their native promoters. The characterized spirochete clones and antibodies generated were then used to conduct functional studies as described below.

### Recognition of TprD and TprK on the surface of *B. burgdorferi* by IFA using antibodies generated against recombinant proteins

**Figure 2:**
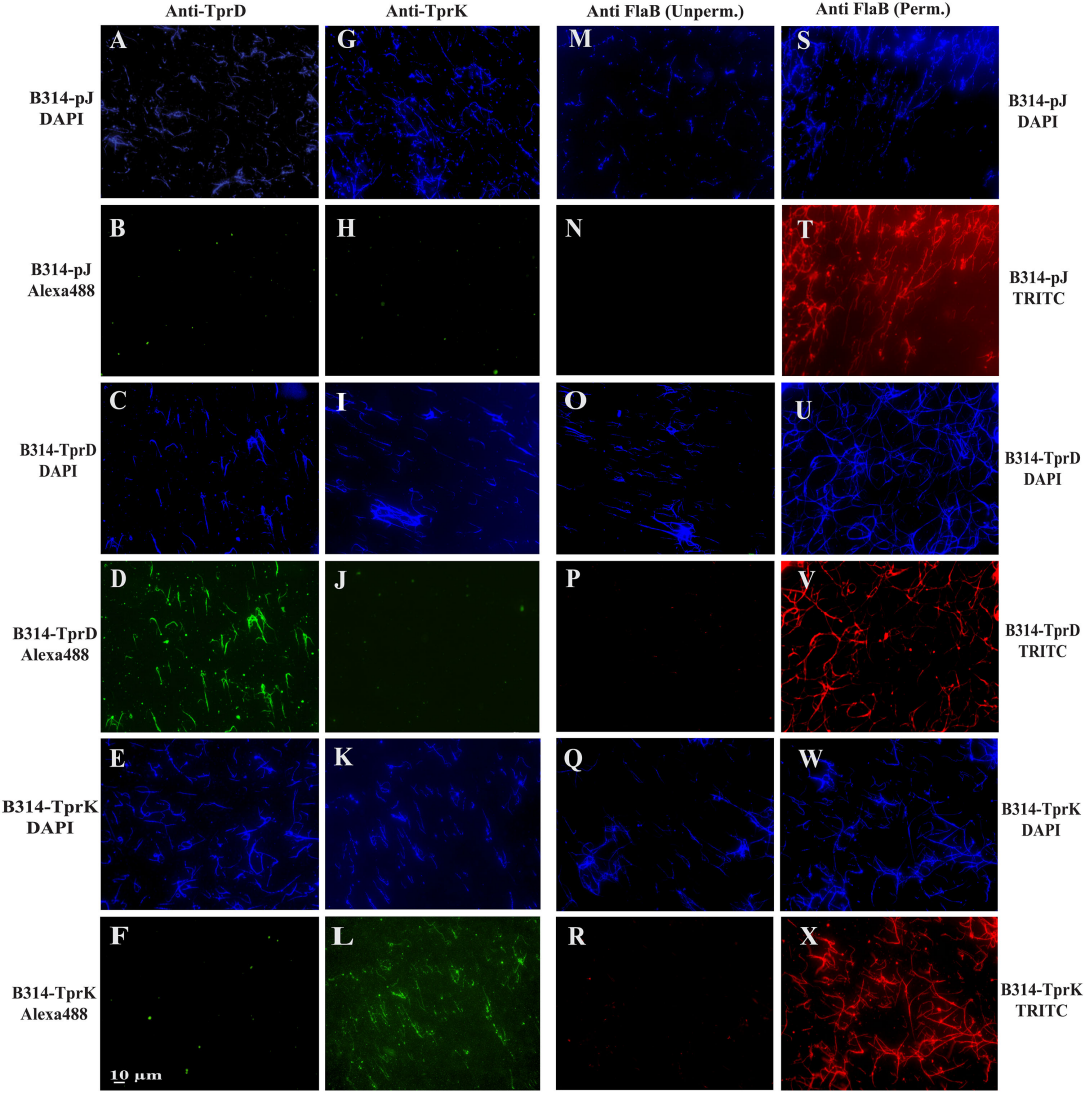
Detection of surface expression of TprD and TprK in *B. burgdorferi* B314 strain. IFA was performed to determine if TprD and TprK are located on surface of spirochetes. To verify membrane integrity, anti-FlaB antibodies that recognize periplasmic proteins were used. Panels **(A–F)** show staining with anti-TprD antibodies, **(G–L)** with anti-TprK antibodies. After washing, both were followed by treatment with Alexa Fluor 488 conjugated secondary antibodies exhibiting green fluorescence. B314(pJ) was used as a negative control. Panels **(M–X)** controls show treatment with anti-FlaB antibodies followed by TRITC conjugated anti-mouse antibodies, with or without permeabilization of spirochetes prior to primary antibody treatment. Bar indicates 10 μm.

We designated TprD and TprK expressing B314 strain as B314-TprD and B314-TprK, respectively in **Figure 2**, while the strain transformed with the pJSB175 vector alone is referred to as B314-pJ and served as a negative control in various experiments. IFA was conducted three times using two coverslips for each treatment per experiment, and a total of six images were collected from different experiments. Microscopic fields for imaging were selected where ~150 spirochetes could be detected by DAPI staining. Surface labeling of B314-TprD and B314-TprK with respective antibodies showed specificity of reactivity because B314-pJ strain did not show any reactivity with these antibodies. (**Figures 2D, L** versus **2B, H**). DNA staining by DAPI confirmed the presence of spirochetes in the respective fields. B314-TprD showed strong fluorescence signal with anti-TprD while punctate fluorescence was observed in B314-TprK treated with anti-TprK antibodies. Average of fluorescence intensity measured from six fields for each treatment from different experiments were: 0.068 ± 0.03 for B314-pJ with anti-TprD; 0.065 ± 0.03 for B314-pJ with anti-TprK, 11.68 ± 3.97 for B314-TprD with anti-TprD; and 12.83 ± 1.90 for B314-TprK with anti-TprK. All three strains (B314-pJ, B314-TprD, and B314-TprK) were also probed with antibodies against FlaB protein of *B. burgdorferi* under unpermeabilized condition in parallel. The lack of fluorescence indicates that the spirochete outer membrane integrity was maintained during IFA. A strong TRITC signal detected in all strains after permeabilization validated antibodies recognition of periplasmic flagellar FlaB (**Figures 2T, V, X**). These results demonstrate that the antibodies raised against TprD and TprK specifically recognize the respective proteins on the surface of intact *B. burgdorferi*. This experiment was repeated three times.

### TprD and TprK on *B. burgdorferi* surface are recognized by secondary syphilis patient convalescent and infected rabbit sera

**Figure 3:**
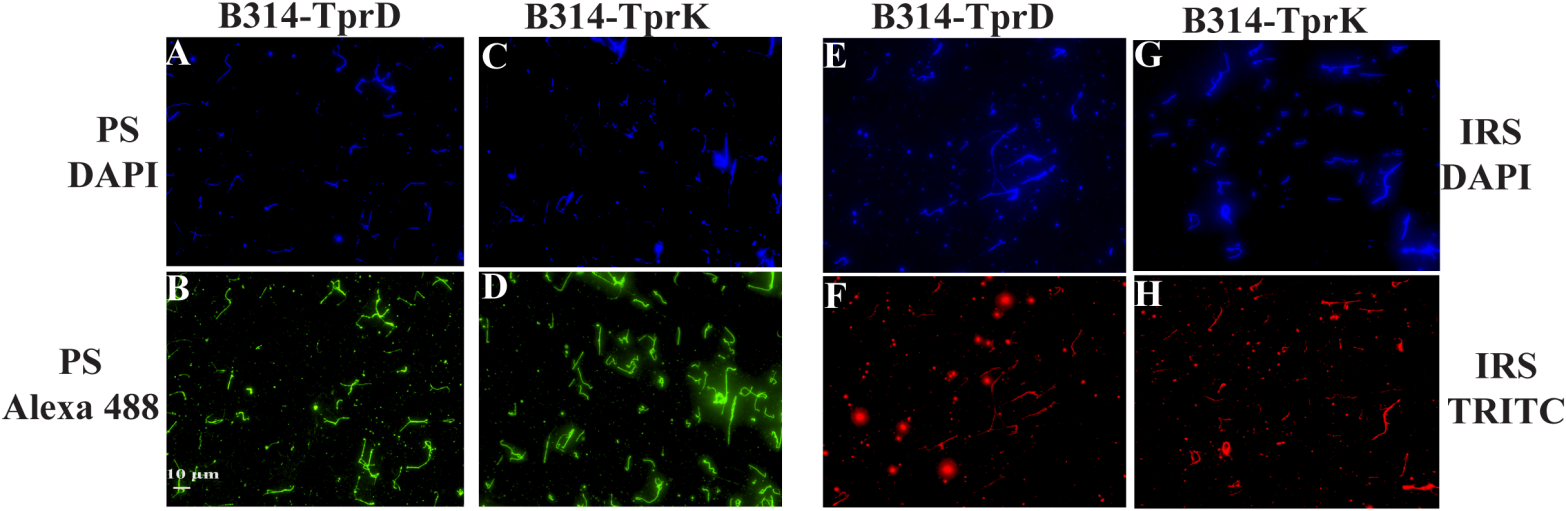
Immunofluorescence analysis using convalescent patient and rabbit sera confirm the expression of TprD and TprK on *B. burgdorferi* surface. Panels **(A, C, E, G)** depict the presence of all spirochetes in the fields by DAPI staining. Spirochetes in **(B, D)** were first treated with secondary syphilis patient serum (PS), and after washing, followed by Alexa Fluor 488 conjugated anti-human antibodies treatment. **(F, H)** panels depict red fluorescence due to TRITC conjugated anti-rabbit antibodies treatment following incubation with infected rabbit serum (IRS). Bar indicates 10 µm.

To further confirm that TprD and TprK are expressed in *B. burgdorferi* and are displayed on the surface, IFA was performed using secondary syphilis patient serum (PS) and long-term (60 days of infection) infected rabbit serum (IRS). Again, staining by DAPI demonstrated the presence and distribution of spirochetes across all samples (**Figures 3A C, E, G**). Surface labeling was observed when patient serum (**Figures 3B, D**) or IRS (**Figures 3F, H**) was used. Together, these results validate the expression of antigenic Tpr proteins and show that they are exposed on the surface of the B314 strain.

### Validation of mouse antibodies generated against chimeric TprD and TprK using *T. pallidum* Nichols and SS14 Strains

**Figure 4:**
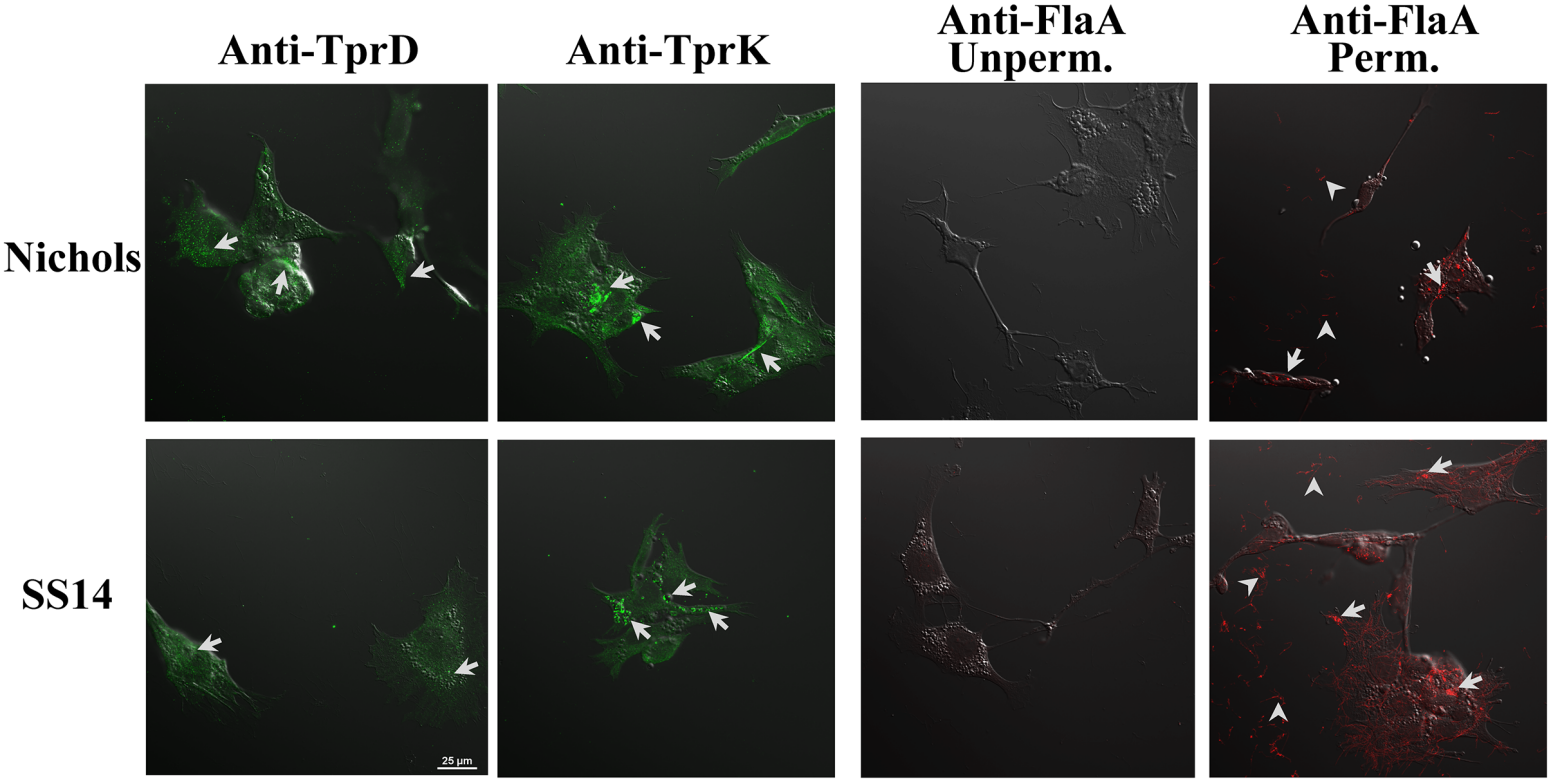
Detection of TprD and TprK on the surface of *T. pallidum* co-cultured with Sf1Ep cells *in vitro*. IFA was conducted with co-cultured Nichols and SS14 strains in chambered slides by incubating with either anti-TprK or anti-TprD antibodies followed by Alexa Fluor 488 anti-mouse antibodies. Unpermeabilized or permeabilized treponemes incubated with anti-FlaA antibodies were followed by TRITC conjugated anti-rabbit antibodies to validate reactivity of the primary antibodies with *T. pallidum* flagellin. Bar indicates 25 µm.

To ensure that antibodies we generated indeed recognize surface *T. pallidum* proteins, we conducted IFA with either Nichols, or SS14 strain co-cultured *in vitro* with Sf1Ep rabbit cells in the chambered slides. IFA revealed differential surface staining patterns for TprD and TprK on fixed Nichols and SS14 strains. Anti-TprK antibodies produced a clear, slightly punctate green fluorescence along the length of treponemes in both strains, indicating surface accessibility of TprK (**Figure 4**). Total TprD and TprK proteins were rather low in both species of spirochetes and poorly detectable when 1:2,000 dilution of primary antibodies was used in immunoblotting (**Supplementary Figure 2**). Overall fluorescence signal appeared lower in *T. pallidum* compared to the surrogate B314 spirochetes expressing TprD and TprK (**Figure 3** versus **4**). Furthermore, relatively weaker staining of SS14 compared to that on Nichols strain could be due to differential surface expression and slight variation in the selected epitopes of TprK, and of TprC/TprD, which is duplicated in the Nichols strain. As an internal control, unpermeabilized treponemes incubated with anti-FlaA antibodies showed no detectable fluorescence in either strain, consistent with FlaA being a periplasmic flagellar protein inaccessible for surface staining. After permeabilization, both Nichols and SS14 exhibited strong TRITC fluorescence, which confirms successful membrane disruption and validated antibodies’ reactivity to FlaA protein.

### Surface labeling of *T. pallidum* strain with secondary syphilis patient serum and infected rabbit serum

**Figure 5:**
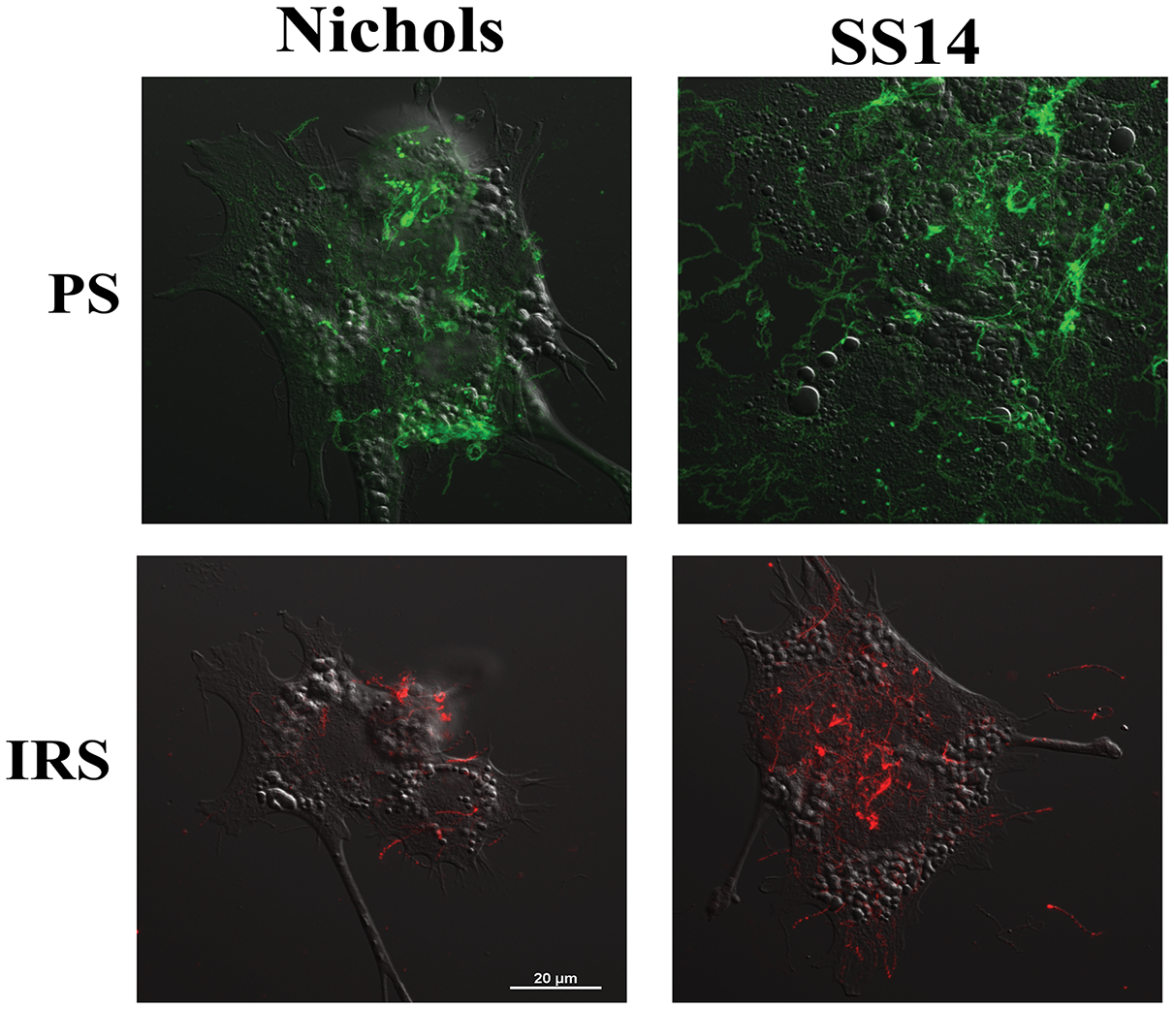
IFA-based detection of surface proteins of *T. pallidum* Nichols and SS14 with secondary syphilis patient serum (PS) and infected rabbit serum (IRS). IFA with PS and IRS followed by FITC- and TRITC-conjugated secondary antibodies, respectively indicates low abundance of overall antigenic proteins on *T. pallidum* surface. Bar indicates 20 μm.

Nichols and SS14 strains labeling with a patient serum (PS) and infected rabbit serum (IRS) was detected by IFA revealing punctate green fluorescence for the syphilitic patient and red fluorescence for convalescent rabbit serum, showing surface exposure of all proteins in *T. pallidum*. Interestingly, the fluorescence using PS was more pronounced than that with IRS. SS14 strain showed higher signal when each primary polyclonal antibodies followed by respective fluorescent secondary antibodies were used (**Figure 5**).

### Evaluation of opsonophagocytosis

Since our antibodies were raised in mice, we used J774A.1 macrophage cell line for opsonophagocytosis experiment. To evaluate the contribution of TprD and TprK in antibody-mediated clearance, we first conducted opsonophagocytosis using the microscopy-based protocol that we previously optimized (**Supplementary Figure 3**). Although some phagocytosis was observed after 3h of coincubation, results were ambiguous likely due to slow opsonophagocytosis documented with *T. pallidum* surface proteins. In fact, we previously showed slow opsonophagocytosis (needed 6h coincubation) even using a surrogate system when another *T. pallidum* protein was expressed (25). Therefore, we further employed a highly novel IncuCyte-based assay to ascertain opsonophagocytosis by J7741.1 cells described below.

### Opsonophagocytosis of B314 strain expressing TprD and TprK by mouse macrophages

**Figure 6:**
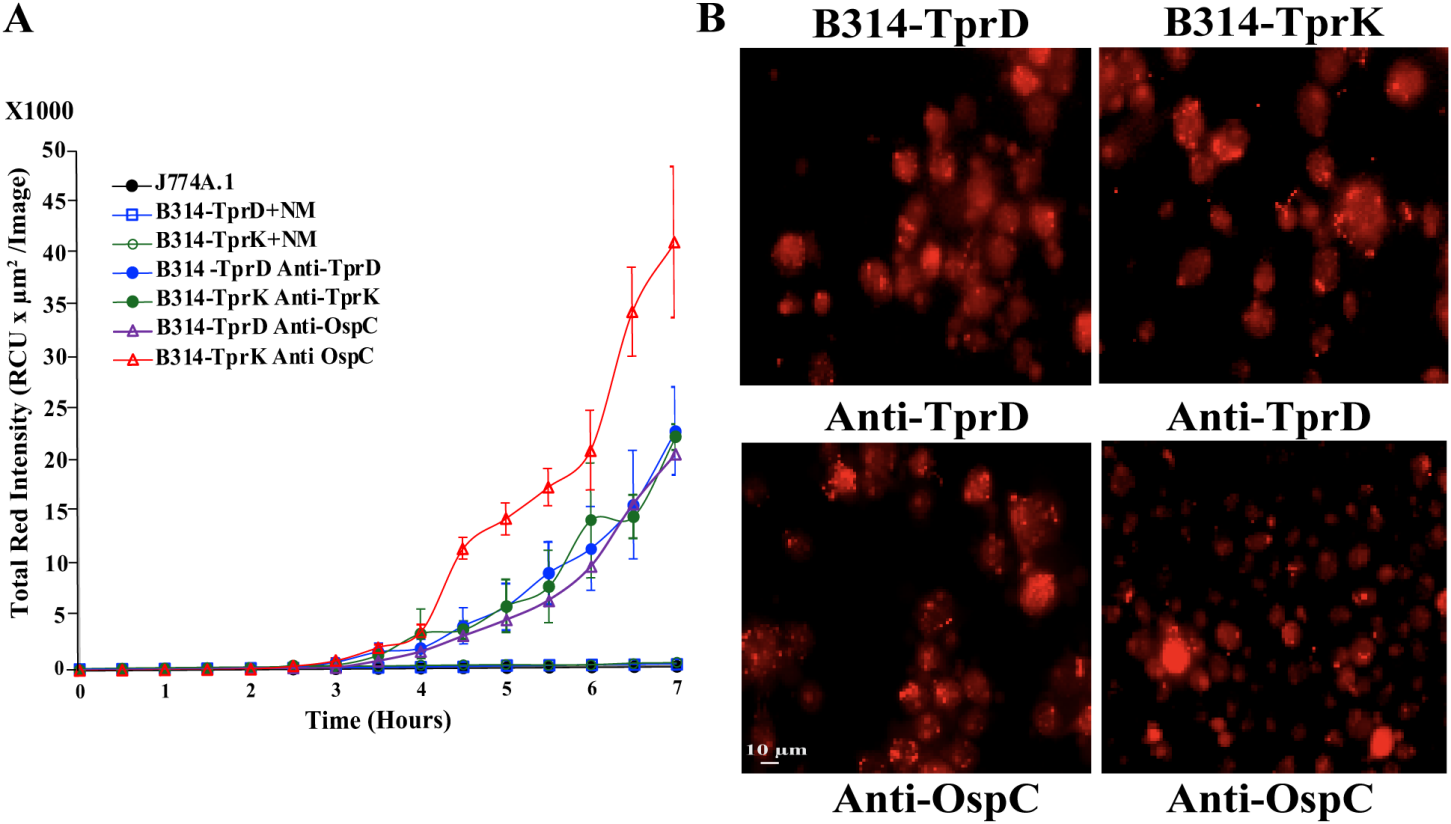
Opsonophagocytosis of B314 strains labeled with anti-TprD and anti-TprK antibodies with pHrodo™ Red, succinimidyl ester using IncuCyte system. **(A)** Quantitative data analysis of opsonophagocytosis show uptake of B314 expressing TprD or TprK with anti-TprD or anti-TprK antibodies over 7h of coincubation. *B. burgdorferi* surface lipoprotein OspC with antibodies against OspC were used as positive control and uninfected Balb/c normal mouse serum (NM) as negative controls. Average ± SEM of data from three wells each is shown. Data from nine pre-formatted points was collected from each well by the IncuCyte instrument. **(B)** Microscopic images show a visual representation of opsonophagocytosis after ~16h (overnight) incubation in the IncuCyte machine. Bar indicates 10 μm.

The IncuCyte system allows longer co-incubation of opsonized live bacteria with macrophages and has provision for automated data collection at selected timepoints. Thus, total red fluorescence generated after pH sensitive dye labeled bacteria enter the acidic phagosome is recorded. In this experiment, B314 strains were preincubated with pHrodo™ Red, succinimidyl ester together with respective anti-Tpr antibodies, and after washing, added to J774A.1 cells in 24-well plate (with and without coverslips). Anti-OspC antibodies were included as a positive control and serum collected from uninfected, normal Balb/c mouse (NM) as negative controls. Detectable fluorescence appeared after 4h incubation and continued to increase at later timepoints only when antisera against spirochete proteins were used and not in the NM controls (**Figure 6A**). In fact, controls were almost indistinguishable from J774A.1 cell alone wells, i.e., exhibiting no fluorescence. Average of data collected from each well (nine points) without coverslip by machine at each time point are shown in this figure. After completion of the opsonophagocytic assay, coverslips from replicate wells were washed, mounted and imaged using Nikon ECLIPSE 90i fluorescence microscope using a 40x objective lens in the TRITC channel to observe phagocytosis throughout the coverslips (**Figure 6B**). Bright red fluorescence observed in most macrophages throughout indicate multiple red puncta in vesicles confirming opsonophagocytosis data obtained by IncuCyte machine. Brightness of the phagosomes in images was more pronounced when multiple bacteria were simultaneously engulfed by a macrophage.

### Opsonophagocytosis of *T. pallidum* strains after treatment with Anti-TprD and Anti-TprK antibodies

**Figure 7:**
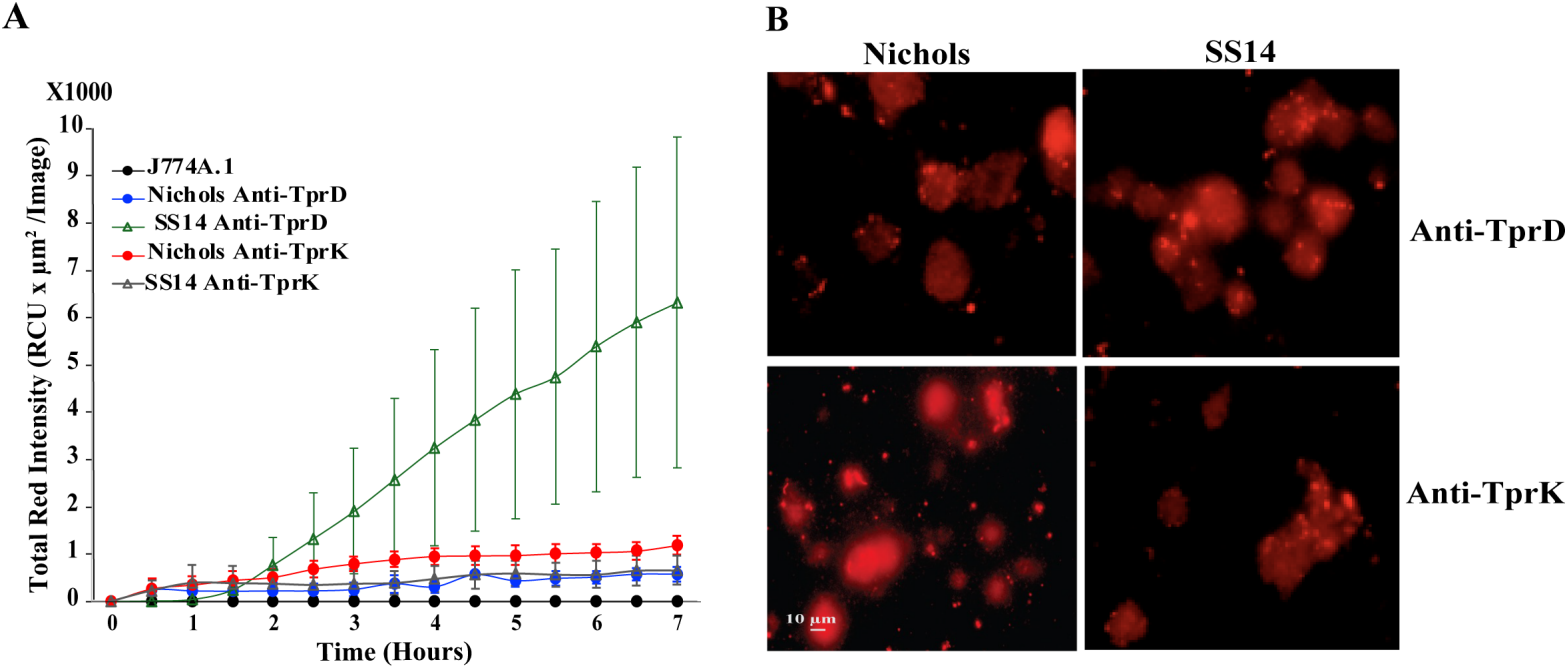
Opsonophagocytosis of *T. pallidum* Nichols and SS14 strains labeled with anti-TprD and anti-TprK antibodies **(A)** Quantitative analysis of opsonophagocytosis showing increased uptake of Nichols and SS14 strains following prolonged incubation after treatment with either anti-TprD or anti-TprK antibodies. Average ± SEM of data from three wells each is shown. Data from nine pre-formatted points was collected from each well by the IncuCyte instrument. **(B)** Microscopic images showing a visual representation of opsonophagocytosis. Bar indicates 10 μm.

Successful outcomes of opsonophagocytosis of *B. burgdorferi* surrogate system incentivized us to conduct a similar assay with *T. pallidum* strains. Opsonophagocytosis of live *T. pallidum* Nichols and SS14 strains as indicated by red fluorescence signal, when reacted with our anti-TprD and anti-TprK antibodies, was relatively lower (**Figure 7A**) than that observed for B314 strain expressing these Tprs (**Figure 6A**). The most pronounced fluorescence signal was obtained with SS14 strain pretreated with anti-TprD antibodies. The imaging of multiple fields treated in parallel on coverslips in the same way in wells of the same plate visually demonstrate opsonophagocytosis of *T. pallidum.* The experiment was conducted two times and representative images from one experiment are shown (**Figure 7B**).

### B314 strain expressing either TprD or TprK gain ability to bind to 293, C6-glioma, and HeLa cell lines

**Figure 8:**
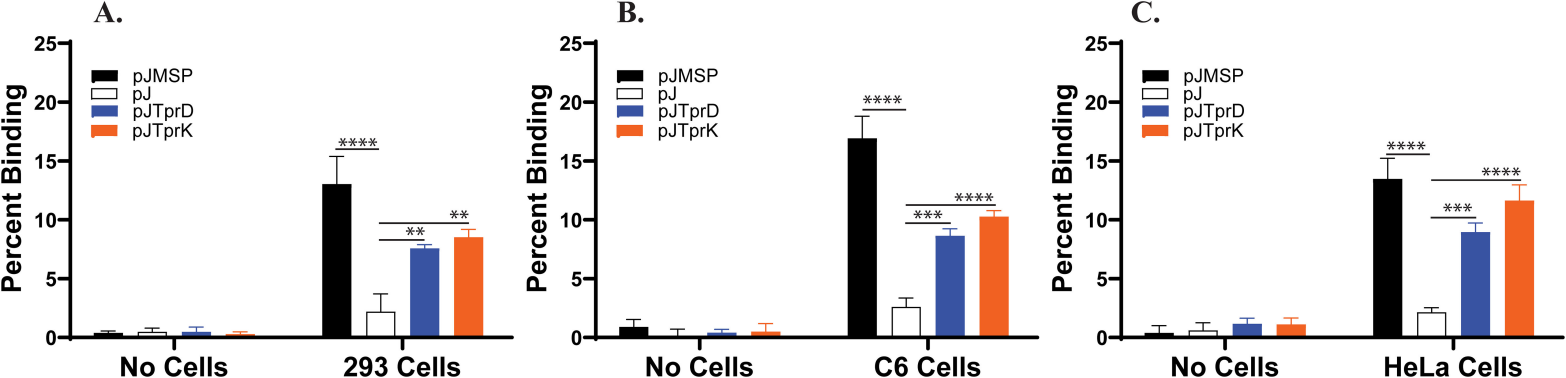
B314 acquires ability to bind to mammalian cells after TprD and TprK expression. Radiolabeled spirochetes carrying the shuttle vector pJSB175 alone (pJ), or those expressing *T. pallidum tprD* or *tprK* genes or possessing the *T. denticola* Msp encoding gene were incubated with; **(A)** 293, **(B)** C6 Glioma, and **(C)** HeLa cell monolayers for 1h. After washing, binding in each well was determined using scintillation counter. Bars represent mean ± SD of three experiments and in each experimental eight replicate wells were used (total 24 wells). Statistical analysis was conducted using data from three independent binding experiments. Statistical significance from three independent experiments was determined using one-way ANOVA with Dunnett’s *post-hoc* test in GraphPad Prism and adherence level by each protein expression group to a cell line was compared to the negative control B314(pJ) binding to the same cell line and p values calculated (**p <0.01, and *** p<0.001, **** p<0.0001).

We used radiolabeled B314 clones carrying either the shuttle vector alone (pJ), or the genes encoding TprD or TprK (**Supplementary Figure 1**). B314 expressing Msp protein of *T. denticola* was included as a positive control. We conducted binding experiments with three different mammalian cell lines that are representatives of cell types possibly targeted by *T. pallidum* during infection. Results are average of three independent experiments, each employing eight replicate wells per treatment with standard deviation shown (**Figure 8**). Statistical analysis included in the figure are from all three experiments. In the No cell control, all constructs showed only background levels of radioactivity indicating that no *B. burgdorferi* bound to the empty wells. B314 with the vector control exhibited minimal binding (~1-3%) to all three cell lines while the expression of TprD or TprK significantly increased binding, reaching ~7-9% for TprD and ~9-11% for TprK, particularly on HeLa cells. The Msp-expressing clone demonstrated the highest level of binding with ~12-13% on HEK293 cells, ~17-18% on C6 cells, and ~13-14% on HeLa cells (**Figures 8A–C**). These results demonstrate that B314 acquires ability to adhere to host cells only when TprD, TprK, or Msp is expressed, supporting roles of TprD and TprK of *T. pallidum* as adhesins.

## Discussion

The re-emergence of syphilis among young adults and the increase in cases of congenital syphilis are serious global health challenges that underscore the need for defining *T. pallidum* molecular pathogenesis mechanisms for development of effective diagnostic and preventative measures. Although some unique *T. pallidum* proteins are now recognized as its virulence factors, significant gaps remain regarding the subcellular location of various proteins, the transcriptional mechanisms of its virulence factors, and most importantly, functional assessment of its proteins that are expressed at significant levels. Electron microscopic examination and cryo-electron tomography previously showed molecular architecture of *T. pallidum* and scant presence of surface proteins (28, 69, 70). For extracellular pathogens like *T. pallidum* and *B. burgdorferi* that cause systemic diseases, receptor-mediated adherence to host cells is critical for organism persistence and long-term survival despite stimulation of a strong adaptive immune response.

The Tpr proteins have been evaluated by different researchers as potential virulence factors because of the presence of predicted N-terminal cleavable signal peptide and the antigenic variation in TprK (46, 47, 52). Among these, TprK is the most extensively studied candidate (26, 51, 52, 63, 71). Detection of faint bands in Western blot could be due to low production of Tprs in both species of spirochetes and poor reactivity with our antibodies. We further decided to investigate the location of both TprK and TprD using robust immunofluorescence and opsonophagocytosis assays. Instead of using recombinant proteins that do not offer conformational integrity of outer membrane proteins, we employed a *B. burgdorferi* surrogate system and gain-of-function approach which we successfully used previously for other *T. pallidum* proteins (25, 34, 36). Since one allele each of TprC/D and TprK were used in this study, epitope differences in TprC/D in different strains and antigenic variation occurring in TprK in *T. pallidum* are not the focus of our study. Labeling of proteins specifically when TprD and TprK are expressed in B314 with antibodies generated against the respective putative surface epitopes chimeras were used indicate that these indeed are surface proteins.

Antigenic variation exhibited by TprK in *T. pallidum* and surface localization of the homologous Msp in *T. denticola* support our finding that Tprs are surface proteins. Furthermore, antigenic variation is exclusively associated with surface-exposed proteins of microbes that cause long-term infection. Antigenic variation allows pathogens to escape specific adaptive immune responses during infection. In this study, only one allele of each protein was used avoiding the influence of variation of TprC/D in different strains and antigenic variation of TprK. Furthermore, both Tpr proteins were recognized on the B314 strain surface by a secondary syphilis patient and a convalescent rabbit serum. Surface labeling also occurred on *T. pallidum* Nichols and SS14 strains grown in co-culture with rabbit epithelial Sf1Ep cells. In general, our results agree with previous demonstration that TprD/K proteins are surface exposed (47). Survival and growth of *T. pallidum* now in co-culture system has moved the field forward in recent years. Although success of co-culture system further indicates importance of interaction of *T. pallidum* for its multiplication, limit of this system is that many experiments cannot be done using this system, such as labeling followed by binding assays that are routinely used for other spirochetes grown in pure culture including *B. burgdorferi*. More extensive studies would be feasible using *T. pallidum* when it would become possible to grow this spirochete in pure culture.

The outer surface molecules of extracellular pathogens, which are often immunogenic, play a major role in adherence and pathogenesis, and in immune system mediated clearance because they are often targets of bactericidal or opsonic antibodies (47, 51). Previous studies have shown that antibodies generated in syphilitic patient serum against outer membrane proteins promote opsonophagocytosis (63, 65, 72, 73), and exhibit complement-dependent treponemicidal activity (74). Macrophage-mediated phagocytosis of opsonized *T. pallidum* is a crucial mechanism for their clearance from primary and secondary lesions (75–77), and opsonic antibodies are essential for killing treponemes by macrophages (62). Our immunolabelling and opsonophagocytosis experiments previously revealed that populations of *T. pallidum* are heterogeneous, consisting of antibody-binding and non-binding subpopulations (25, 51). Moreover, the organisms that bind to antibodies do so with slow kinetics (51, 75, 78). In this study, we used the IncuCyte system to examine opsonophagocytosis when spirochetes were pre-labeled with specific antibodies and a pH-sensitive dye to obtain data after longer incubation. We found that anti-TprD and anti-TprK antibodies, and not NM control serum, promote opsonophagocytosis of surrogate *B. burgdorferi* expressing either of these two proteins as well as of two strains of *T. pallidum* by J774A.1 cells. Thus, our results agree with previous reports which showed that anti-TprK antibodies facilitate opsonophagocytosis of *T. pallidum* (47). These results also support our findings in which immunization with recombinant TprK or *B. burgdorferi-*expressing TprK induced partial protection in rabbits challenged with the *T. pallidum* Nichols strain (35, 47).

We further conducted functional analysis of TprD and TprK proteins. Since recombinant proteins may not accurately represent the surface epitopes of proteins, our related spirochete surrogate system represents adherence mechanism more closely to *T. pallidum*. Tprs are related to the Msp protein of *T. denticola*, which has been previously reported to be surface-exposed and exhibiting functions both as an adhesin and a porin (47). Therefore, we included Msp as a positive control in our binding assays. Our results demonstrated that TprD/K expression confer ability to bind to the host cells by the B314 strain which is otherwise poorly adherent as demonstrated by binding of vector containing B314(pJ) strain (**Figure 8**). Our results suggest the role of TprD/K in tissue colonization during infection, potentially in skin lesions and/or during neurosyphilis manifestations (71, 79). Recently, Molini and colleagues (61) observed cross-reactivity of antibodies toward variant peptides of TprC, TprD, and TprD2 in their quest to identify B-cell epitopes across TprC/D variants which also incentivizes exploration of these Tprs as vaccine components. Furthermore, testing of a multiple recombinant protein-based vaccine that combines the NH_2_-terminal fragment of TprK and the NH_2_-terminus fragments shared by Tpr Subfamily 1 proteins, together with Tp0751, is currently in progress in other laboratories (80).

To summarize, one major limitation of the present study is the inability to conduct experiments with physiologically active *T. pallidum.* Consequently, we used a surrogate system to determine how adherence is affected by *T. pallidum* TprD and TprK. Future studies with physiologically active *T. pallidum* and primary human tissues could lead to better understanding of the mechanisms and roles of adherence-mediated colonization of host tissues during infection. The results of this study emphasize the importance of surface-exposed TprD/TprK antigens as targets for antibodies which facilitate opsonophagocytosis. Our findings further support the inclusion of TprD and TprK in future vaccines for protection against *T. pallidum* infection. Thus, a multivalent vaccine that includes antigenic epitopes of Tprs offers high promise of development of an effective and successful vaccine against syphilis in the future.

## Material and methods

### Ethics statement

The *T. pallidum* Nichols and SS14 strains were grown in *in vitro* co-culture system in the Giacani lab according to the Edmondson et al. protocol (81) in chambered slides. Co-cultured *T. pallidum* were fixed with 4% paraformaldehyde made in PBS for IFA. However, *T. pallidum* recovered from infected New Zealand White rabbits were used for opsonophagocytosis experiments. Both IFA and opsonophagocytosis experiments were conducted in Parveen’s laboratory. All procedures followed the standards outlined in the Guide for the Care and Use of Laboratory Animals under the protocol reviewed and approved by the University of Washington Institutional Animal Care and Use Committee (IACUC protocol 4243–01; PI Lorenzo Giacani). Polyclonal antibodies against recombinant putative surface exposed loops of TprK and TprD were generated in BALB/c mice in Parveen’s laboratory using our previously established immunization procedure. These experiments were performed under Parveen’s PROTO202000087 protocol which was approved by Rutgers Biomedical and Health Sciences IACUC. De-identified secondary syphilis patient serum used in this study was generously provided by the late Dr. Centurion-Lara.

### Cloning of genes for *T. pallidum* proteins expression

One allele of both TprD and TprK encoding sequence with their native promoters were cloned in *B. burgdorferi* shuttle vector, pJSB175. To avoid issues with overexpression of proteins under inducible promoters, we included upstream and downstream regions from open reading frames because promoter regions of these *T. pallidum* proteins are not yet defined (**Supplementary Figure 1**).

### *B. burgdorferi* strains and culture

*B. burgdorferi* strains were cultured in homemade 1X Barbour-Stoenner-Kelly (BSK II) medium containing 6% rabbit serum BSKII+RS at 33°C. Transformation and selection of clones was conducted as previously described with slight modifications (82). The high passage, non-infectious, non-adherent *B*. *burgdorferi* B314 strain was used for transformation with pJSB175 shuttle vector alone, or with Tprs for the expression of TprD, and TprK of *T. pallidum* under their native promoters. Vector transformed strain was used as negative control in various experiments. Briefly, electrocompetent *B. burgdorferi* strain B314 was transformed with 30 μg of each plasmid, and bacteria were allowed to recover overnight in BSKII+RS medium in the absence of antibiotic selection. The bacteria were then plated on BSKII+RS agarose medium containing 100 µg/mL streptomycin. Three clones for each transformed colonies were recovered and characterized one clone from each transformation was used in this study. Transformed clones were grown in liquid BSKII+RS medium with 100 µg/mL streptomycin and cultures grown until mid-logarithmic stage (~1x10^8^ spirochetes per mL) used for different experiments.

### TprC/D and TprK putative surface epitopes chimeras used for antibody production

To generate polyclonal antibodies targeting the putative surface exposed domains of the TprC/D protein family identified previously (83), DNA constructs in pET30a vector were custom-designed and synthesized by GenScript (**Figure 1**). They were used to express recombinant chimera with defined Major Outer Sheath Protein N-terminal region (MOSP^N^), the Central Variable Region (CVR), and the C-terminal MOSP^C^ β-barrel domains. We also designed clones in pET30a to produce chimeric protein that includes six variability domains of TprK (3). For Isopropyl β-D-1-thiogalactopyranoside (IPTG)-induced production of each polyhistidine tagged (His-) proteins, *E. coli* expression strain, BL21(DE3)pLysS was used and proteins purified using Nickel-affinity column following manufacturer’s instructions (Novagen). Purified proteins were subsequently used to immunize five Balb/c mice each to generate polyclonal antibodies as we described previously (84).

### Immunoblotting and Enzyme Linked Immunosorbent Assay for titration of anti-TprD and anti-TprK antibodies

The pellet of B314 harboring designated plasmids were resuspended in 1x protein loading buffer and boiled for 5 minutes. The extract from ~1-2x10^7^ spirochetes were loaded in each lane of 10% SDS-PAGE gel and proteins resolved by electrophoresis. After SDS-PAGE, gels were either stained with Coomassie stain or three parallel sets transferred to Immobilon-P membrane from Millipore for Western blotting with the respective antibodies at 1:2,000 dilution. In addition, pooled antisera from five mice each were titrated against full length His-tagged TprD and TprK by ELISA using 1:50 to 1:6,400 dilutions.

### Indirect Immunofluorescence Assay

IFA was performed to determine whether TprD and TprK are expressed on the surface of *B. burgdorferi* B314 strain transformed with clones generated in pJSB175 shuttle vector (sequence in **Supplementary Figure 1**). Spirochetes were harvested by centrifugation at 8,000 rpm for 10 minutes followed by three washes in PBS containing 0.2% BSA under identical centrifugation conditions. The pellet was then resuspended in PBS/0.2% BSA to achieve a concentration of approximately 2.5–5 × 10^7^/mL. Sterile 12-mm round coverslips were placed into 24-well plates, and each well was overlaid with 400 µL of HBSC buffer (25 mM HEPES, 150 mM NaCl, 1 mM MgCl_2_, 1 mM MnCl_2_, and 0.25 mM CaCl_2_, pH 7.8) supplemented with 0.2% BSA and 0.1% glucose, followed by the addition of 100 µL of spirochete suspension. Plates were centrifuged at 2,000 rpm for 10 minutes at room temperature to promote organisms attachment to the coverslips. After removal of supernatants, spirochetes were fixed with 3% paraformaldehyde for 1h at room temperature and washed three times with PBS. Both unpermeabilized and methanol permeabilized (kept at −20 °C) spirochetes were blocked with PBS containing 5% BSA and 5% heat-inactivated goat serum (blocking buffer) for 1h. Pooled polyclonal antibodies from five mice each generated against TprC/D or TprK putative surface epitopes were used at 1:100 dilution with anti-FlaB monoclonal antibodies against periplasmic protein used as control to ensure integrity of outer membrane. Plates were incubated at room temperature for 2h with gentle rocking. After washing with PBS, all wells were permeabilized again with cold methanol for 10 minutes for DAPI staining (10 µg/mL final) together with Alexa Fluor 488 or TRITC-conjugated secondary antibodies diluted at 1:100 for 1h at 37 °C in the dark and then washed. Coverslips were mounted onto glass slides and examined using Plan Apo λ objective in Nikon ECLIPSE 90i microscope. Similarly, IFA was conducted with *T. pallidum* strains grown in co-culture system in chambered slides, except that a polyclonal antiserum against *T. pallidum* FlaA protein of (generously provided by Dr. Diane Edmondson) was used as a negative control. Due to the presence of Sf1Ep cells in these slides, images were collected using Nikon Ti2 microscope illuminated using a Lumencor Spectra X light engine and images captured with a Hamamatsu ORCA Flash 4.0 V3 sCMOS camera and Nikon NIS Elements software. In each experiment, two coverslips were used for each treatment. The experiment with *B. burgdorferi* B314 strain was conducted three times independently. Microscopic images from a total of six fields were collected from different experiments and fluorescence intensity using image J software in each field of view measured to determine the average after anti-TprD or anti-TprK antibodies treatment. Fields of view with ~150 spirochetes (as determined in DAPI staining) were selected for imaging.

### Opsonophagocytosis assays

We conducted microscopy-based opsonophagocytosis assay (**Supplementary Figure 3**) as described before except spirochetes-macrophage coincubation was for 3h (25). In the second approach, J774A.1 macrophages, cultured in DMEM supplemented with 10% FBS, were plated into 24-well, clear-bottom plate with or without coverslips. After washing to remove culture medium, spirochetes were incubated with primary antibodies/normal mouse serum together with pH sensitive pHrodo™ Red, succinimidyl ester in carbonate buffer (prepared to a final concentration of 1 µM) for 1h in the dark, washed and resuspended in PBS/0.2% BSA. Immediately before starting the assay, spirochetes were added to J774A.1 cells and plates were transferred to the IncuCyte ZOOM imaging system maintained at 37 °C with 5% CO_2_. An acidic milieu in the late phagosome of macrophages promotes red fluorescence emission by pHRodo dye which can be recorded in real time by the IncuCyte machine (85). Nine images per well were acquired every 30 min for 7h using a 10× objective, with an 800-ms exposure for the red fluorescence channel. Image analysis was performed using IncuCyte Basic Software. Phase-contrast segmentation was achieved by applying a cell-specific mask to exclude background regions, and an area filter was used to eliminate objects smaller than 100 µm². Red-channel background noise was corrected using the Top-Hat method with a 100 µm radius and a threshold of 2 corrected units. The assay wells with coverslips were washed and coverslips were mounted and observed using fluorescence microscope as described above.

### Binding assays

Three independent binding assays were conducted using B314 expressing TprD, TprK of *T. pallidum* or Msp of *T. denticola* as previously described (84). Eight replicates were used for each treatment. B314 with vector pJSB175 was included as a negative control for each cell line. Three different cell lines were used in these experiments: HEK293 (a human embryonic kidney epithelial cell line), C6 (a rat glioma line), and HeLa (a human cervical cancer epithelial line). Cell lines available in Parveen’s laboratory were selected to represent tissues colonized by *T. pallidum* during human infection.

### Statistical analysis

All binding experiments were performed independently three times. Eight replicates were used for each treatment. Thus, the values shown in the figure represent the mean ± SD of three independent experiments, each with eight replicates per treatment (total 24 wells). Statistical significance was determined from three experiments using one-way ANOVA with Dunnett’s *post-hoc* test in GraphPad Prism, comparing each group to the negative control B314(pJ) on each cell line.

## Data Availability

The original contributions presented in the study are included in the article/**Supplementary Material**. Further inquiries can be directed to the corresponding author.
