# Supplementary material for: Treponema pallidum TprD and TprK are adhesins and their surface expression promotes spirochetal opsonophagocytosis
Source: Front Immunol. 2026 Mar 6;17:1783902. doi: 10.3389/fimmu.2026.1783902 (PMC12997132; doi:10.3389/fimmu.2026.1783902)
Supplement: Supplementary file 1 [file DataSheet1.pdf]

pJTprD clone from *Treponema pallidum* strain Bal3 for B314 transformation

AAGAGGTTCAGGAAGCAACGGGAACGGAGCACAGTGCGTCCACCTGAAGGAAGAGAATGTACACGATCAA
ACCGGCTGGCATGAGAAGGGCTCCACCCCGAAAGGCAGCGCCGAGCAGTATCGTAGTACGATCGGCCGCT
GGGCAGTGAGCGGTATTTACGTAATCAAAAAAAGCACTAGCGGTGGGCGGGGAAAGCGGAGCACCTCAAC
AGACTGCGAAAGACCAGACCTCTACGTGGCGGTGGGAGACACGAACGATACGTACACCGGGCTGTGGAGG
TTTGACTCCGCCGCGCAAAAGTGGAACCGCGAATGAGTGGCTCTAACCCTACGCTTCCCCTACTCCCGCG
CGTACGGCGGGGCAGGCGTAGGGCGTAATTTTGAAAAATCGAGCAGATTCTCAGTACAAAAAGAGGGTAT
AGGTGCGCCGGGTTCTGCGCGTACCGCTTTGCAGTTCAAATTGTTTTTGCTTTCCCGCCTCTTTTTTATT
TTTACGTCACATATTCCCTAGACGGGTGGGGGGGGGTGAGGTAGAAGTGAGAGGAGGGGGAGTGA**GTG**GG
CAGGCAGGTGATGCAAGCGGGGGTACTTGCGGGCATGGTATGTGCTGCTTCTGGTTATGCAGGCGTACTC
ACTCCGCAGGTCAGTGGCACAGCCCAGCTCCAGTGGGGCATTGCGTTCCAGAAGAATCCACGCACTGGCC
CGGGCAAGCACACCCATGGGTTTCGCACTACCAATAGTCTGACTATTTCCCTGCCGTTGGTGTCAAAGCA
CACCCACACCCGCCGAGGGGAGGCACGCTCAGGGGTGTGGGCACAGCTGCAGCTGAAGGACCTGGCAGTA
GAGCTTGCGTCTTCTAAAAGCTCAACGGCCCTGTCCTTTACCAAACCTACCGCTTCCTTCCAGGCAACCC
TGCACTGTTATGGGGCCTACCTGACAGTGGGTACCAGTCCTTCCTGTGTGGTTAACTTTGCCCAGCTGTG
GAAACCCTTTGTCACCCGTGCCTATTCAGAAGAGGACACTCGCTATGCCCCTGGTTTCTCCGGCTCCGGG
GCAAAACTCGGCTACCAGGCCCACAATGTGGGAAACAGCGGAGTAGATGTGGACATCGGTTTCCTCTCCT
TCCTTTCCAATGGTGCCTGGGATAGTACTGACACCACGCACAGCAAGTATGGCTTCGGGGCCGATGCAAC
GCTTTCCTATGGCGTCGACCGTCAGCGGCTGCTTACGTTGGAGCTGGCAGGGAATGCCACACTGGACCAG
AACTACGTTAAGGGTACCGAAGACTCCAAGAACGAAAACAAAACAGCACTCCTGTGGGGAGTAGGAGGCC
GACTCACCCTCGAACCAGGCGCCGGCTTCCGCTTCTCCTTCGCCCTCGACGCCGGTAACCAACACCAGAG
TAACGCACAATTCTACGCTAGAATGGCTCCCTCACAGAGGGTCCATGAAGTCATCACTAGTCTTGGGGAC
ACGCTGCTGACCTCCCCGCAACAAGATGTTGTTTCATTCTTTGTGCAAGAACTGAGCAAAGGCAGTCTTC
TGGAGAAAGCTGGCTTAGTAATGCTCTTGGCGCAGCGCACCATCGTCGGCTTAGCGTCAAGCGGTGGTTA
CCTAAGACATCTGAATGGCAAAGGCCTAGAAATAAACATGAGGCTCATAGAGCAGCAGAAGAATCCTGAC
GCGCGGATGCGGACAGCACTCTTTATTTCCTGGTTGCAATTCACGTACACCAAAACGCTCAACATAGACG
CGCTCCTGCGTATGCAGTGGAGGTGGCTCTCTTCTGGCATATACTTTGCCACCGCAGGCACTAATATCTT
TGGAGAACGTGTTTTCTTTAAGAATCAAGCAGATCACTTTGATTTTGCCGGATTCCTCAAACTCGAAACC
AAAAGCGGTGACCCCTACACCCACCTGCTCACCGGCCTGAACGCCGGCGTCGAAGCACGCGTGTACATCC
CCCTCACCTACATCTTTTACATAAATAACGGAGGTGCGCAGTACAAGGGAAGTAATTCGGACGGCGTCAT
CAACACGCCTATCTTGAGCAAAGCGTGGTGCGGCTATCGCATCCCCCTCGGTTCCCACGCCTGGCTTGCA
CCACACACATCCGTGCTATGGGCAACAAACCGCTTCAACCACAACCAGAGCGGGGATGCGCTCCTGCGTG
AGCACGCGCTCCAGTACCAGGTGGGACTGACGTTCAGTCCCTTCGAGAAGGTGGAGCTCAGCGCCCAGTG
GGAACAGGGCGTGCTTGCTGACGCTCCTTACATGGGCATTGCCGAGAGCATCTGGTCCGAACGCCACTTC
GGCACCCTTGTCTGCGGAATGAAAGTGACATGG**TAA**AAGCGCGTGCACTCCCCGTGCGTTAGCGGCTATG
CCACAGCCCGAGCCGGTGCCGACATCAACTTCCCGGTGTGGGACGTCCGCCCGCATCGAAGGCCAGCAAT
GTGTTTAAAGACGTCTTTCTCACCAATGCCATGGACATGCAGACGCACGACTGCGCACGCTCCATGGGGC
ATAAGAAAAACGGAGCGAATGGCGACATAGGCGCAGACTGTGTGAGTGTGGAGACCCGTTCAGCTTCTCA
GGCGATGCCGTGGGCATTGCATCTGAAACTGGTGGCGCCGGAGCCCTCAGGCAAGGAGGCACACATTAAG
GGACAGACGCGACCTGCCTCATACCGACTGGGAGGGCAAAGACAGTCAGGGCAAGGCCCCAGCAGGAACT
CCCAGCACGTACGGCGGTACGAACAAAAAGGCCACGCCCCCTGCTGCTCCTGCTGCTCCTACGAAGT

pJTprK clone from *T. pallidum* 8004 strain for B314 transformation

CCCCCAGTTGCAGCACTATGCACGGGCGGTGCAGGATCTCTTCTCGGACCACCCGGTGACGGCCTTTCTG
TATTACCTCCGAACCGGGCATGAATTTTCTTTGGAAGCGTTAGAATCTCATTTTCTGAAAAAAAACGCAG
TTCCGGATTCTGAATGATTGACCCATCTGCCACTTCCCGGTATGGTTCCCCACGTTTAGTTAGTAATGGT
TTTCGGCATCGGAGAAAA**GTGGTG**TATCAGCGGGTAGGGCACAGGCGATTTTCTCTCATTTTCTTTTTCG
TTGTGGTTCTGGGGCGGTCCCCGCGGCTGTGGGCTCTGGTTTCGTTCACCCCGGATATTGAAGGCTATGC
GGAGCTGGCCTGGGGCATTGCATCCGATGGTGGCGCCCTCAAGCATGGATTTAAGACTACTACTGATTTT

AAGATTGTGTTCCCCATTGTGGCAAAGAAGGATTTCAAGTACCGCGGTGAGGGGAATGTCTATGCGGAAA
TTAATGTTAAAGCGTTGAAGTTGAGTTTAGAGTCAAATGGTGGAGCAAAGTTTGACACGAAGGGTTCTGC
AAAGACGATAGAGGCAACCCTGCACTGTTATGGGGCCTACCTGACCATTGGGAAGAATCCTGATTTTAAG
TCAACGTTTGCTGTTTTGTGGGAGCCGTGGACCGCGAATGGGGATTATAAGTCTAAGGGAGATAAGCCGG
TGTATGAGCCGGGGTTTGAGGGAGCCGGGGGAAAGTTAGGGTATAAACAGACTGACATCGCCGGCACGGG
GCTCACGTTTGATATTGCGTTTAAGTTTGCGTCTAACACCGACTGGGAGGGCAAAGACAGCAAGGGCAAC
GTCCCAGCAGGAGTAACCCCCAGCAAGTATGGATTGGGGGGAGATATTTTGTTCGGCTGGGAGCGTACGC
GTGAAGATGGCGTGCAGGAATACATTAAAGTGGAGCTCACCGGCAACTCCACACTGTCTAGCGACTATGC
CCAAGCCCGAGCCCTGGCAGCCGGGGCTAAGGTGAGTATGAAGCTTTGGGGTCTGTGTGCTCTGGCTGCT
ACAGACGTGGGGCATAAGAAAAACGGAGCGCAGGGCACCGTAGGCGCAGATGCGTTGTTGACGTTGGGGT
ATCGTTGGTTCTCGGCGGGAGGATATTTCGCATCGCAGGCCAGCAATGTATTCGGGGGAGTATTTCTCAA
CATGGCCATGCGAGAGCACGACTGTGCTGCCTATATTAAGCTCGAAACCAAGGGGTCTGATCCTGATACT
TCTTTCCTTGAGGGTCTTGATTTGGGTGTTGATGTGCGTACGTACATGCCTGTCCATTACAAAGTCCTAA
AAGCCCTACCCCCAGCCGACATCCACTTCCCGGTGTATGGAAAAGTCTGGGGTTCGTATCGTCATGATAT
GGGTGAGTATGGTTGGGTTAAAGTGTATGCAAACTTGTACGGCGGTACGAACAAAAAGGCCACGCCCCCT
GCTGCTCCTGCTACGAAGTGGAGCAAGGAATATTGTGGGTATTACGAGTGTGGGGTAGTGGTCAGTCCGT
TAGAGAAGGTGGAGATTCGGCTGAGCTGGGAGCAAGGCAAGCTACAAGAGAACAGCAATGTAGTGATAGA
GAAGAACGTGACGGAGCGTTGGCAATTCGTAGGGGCATGTCGCTTGATTTGG**TAG**GGATGTATGGCTCTT
TTCTTTCCGAAGGGGCGAATTTTTGCGCCGGCGCATATCTGGCCGGGCATGACACGCAGCGGAGGTAGGC
GGGGTGTGTAGACGTTTGTGCCATCACAGGTGGCGCGTGTGGGGAAAGGTTGCTTCCCTGGGAGTGCTCC
TTCTAGGAGGGCTTGTTGCCTGCACTTCAAGCGCAGCCGGGTCAACCTCCAACACGCGGCCGGGGGTGCG
TATGACGATCACCAGCCGCTACCCCTTCGATCGCACTATGCAGCTTTTGGAGAGCGCTTTGCGCACGCAG
GGCTTTAGCGTTTTTGGTATTGTTGACTACCGCGA

Fig S1. *T. pallidum* TprD and TprK genes with flanking sequences cloned in pJSB175 shuttle vector of *Borrelia burgdorferi*. Sequence of Tpr clones used for transformation of *B. burgdorferi* non-infectious B314 strain to express TprD and TprK. In both clones, putative start codon is marked by bold blue font (underlined codon proposed previously as the potential start codon) and stop codon is marked by bold black font. Upstream sequence including promoter (not yet defined for these two proteins) to express genes under their native, *T. pallidum* promoters in *B. burgdorferi* and sequence downstream of both open reading frames are also provided.

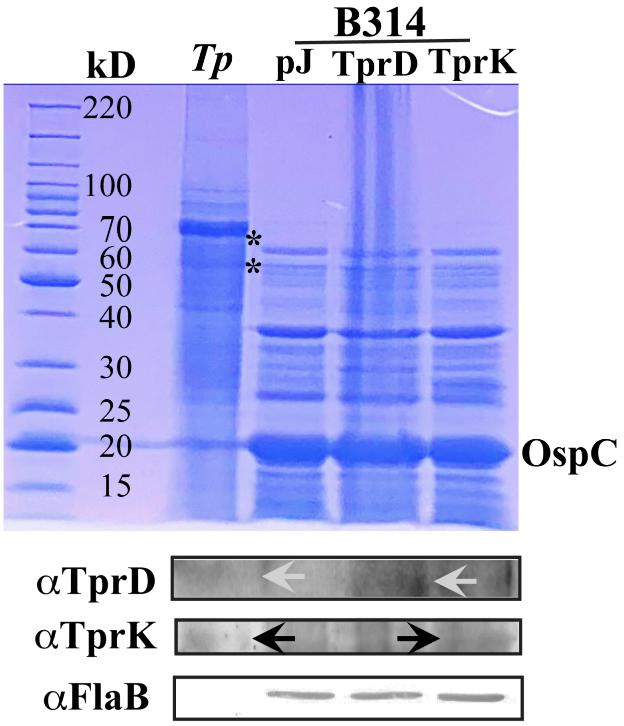

Fig S2. Coomassie staining and immunoblotting of total protein extract resolved by SDS-PAGE. Relatively low-level production of TprD and TprK (marked by asterisks in Coomassie stained *T. pallidum* lane) and detection of faint bands in Western blot analysis of B314 expressing these proteins were observed. Monoclonal antibodies against FlaB of *B. burgdorferi* do not recognize *T. pallidum* flagellin protein.

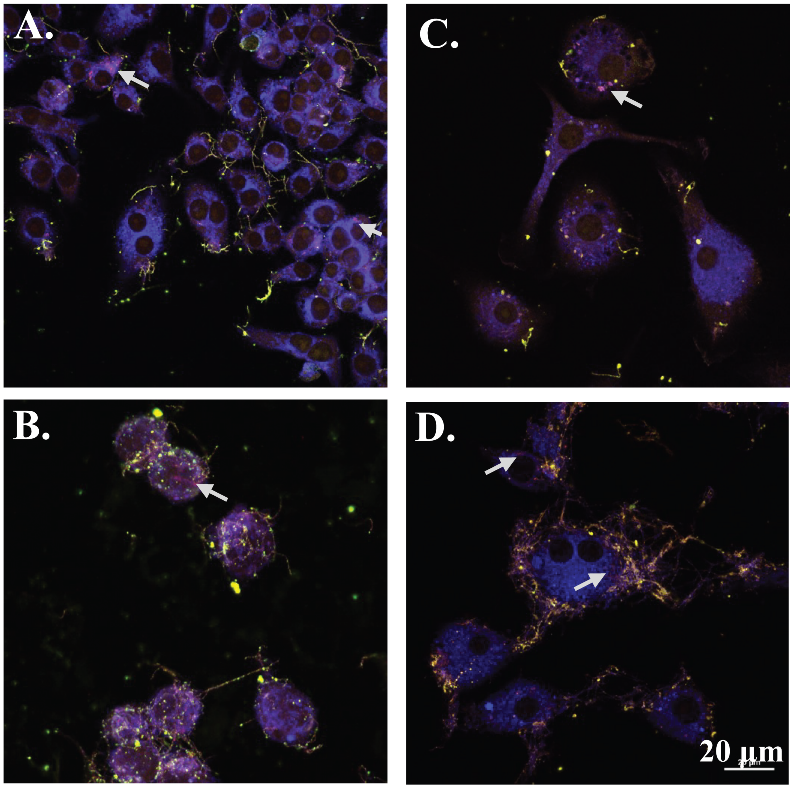

Fig S3 Detection of antibody-mediated phagocytosis of *Borrelia burgdorferi* surrogate strains by mouse macrophages.

The *B. burgdorferi* surrogate strain B314 expressing TprK (B314pJTprK; panels A and B) or TprD (B314pJTprD; panels C and D) was incubated with anti-TprK, anti-TprD, or anti-OspC antibodies and subsequently added to the mouse macrophage cell line J774A.1. After incubation at 37°C, cells were fixed, differentially stained, and examined by fluorescence microscopy. Extracellular spirochetes were stained with the respective antibodies prior to permeabilization and appear green (appearing yellow due to channel overlay), whereas intracellular (phagocytosed) spirochetes were labeled following permeabilization and appear red. Nuclei were counterstained with DAPI (blue). Panels A and C show staining with anti-TprK and anti-TprD antibodies, respectively, while panels B and D show staining with anti-OspC as a positive control for opsonophagocytosis. Arrows indicate representative phagocytosed spirochetes.

Table S1: Antibodies used.

Antibodies	Type	Animal used	Source	Catalog No.
Anti-TprD Outer loops	Polyclonal	Mice	Generated by the lab	NA
Anti-TprK Surface	Polyclonal	Mice	Generated by the lab	NA
Anti-FlaB (Borrelia)	Monoclonal	Mice	From Dr. Erol Fikrig	NA
Anti-FlaA (Treponema)	Polyclonal	Rabbit	From Dr. Edmondson	NA
Anti-mouse AlexaFluor 488	Polyclonal	Goat	Invitrogen	A10684
Anti-mouse TRITC	Polyclonal	Goat	Sigma	T5393
Anti-rabbit TRITC	Polyclonal	Goat	Invitrogen	A11013
Anti-human AlexaFluor 488	Polyclonal	Goat	Invitrogen	A11013
